# Dissecting the Mechanisms of Apolipoprotein E, Blood‐Brain Barrier, and Amyloid Beta Provide insights into Alzheimer's Disease Pathogenesis

**DOI:** 10.1002/alz70855_101337

**Published:** 2025-12-23

**Authors:** Musa Omoyine Iliyasu, Abayomi Ajayi

**Affiliations:** ^1^ Prince Abubakar Audu University (Formerly known as Kogi State University), Anyigba, Kogi State, Nigeria; ^2^ Prince Abubakar Audu University, Anyigba, Nigeria, Anyigba, Kogi State, Nigeria

## Abstract

**Background:**

Alzheimer's disease (AD), a progressive neurodegenerative disease, is the most common type of dementia affecting people over 65 years of age. The prevalence of AD is rising quickly because of extended lifespans, and by 2050, there will be roughly 115 million AD patients worldwide. Apolipoprotein E (APOE), the major genetic risk factor of AD, has been reported to mediate blood‐brain barrier (BBB) integrity and influence amyloid beta aggregation. Findings have initiated the future development of APOE‐targeted AD therapeutics. The present study aimed to review the latest developments in APOE, BBB and amyloid beta mechanisms, mediating AD pathogenesis.

**Method:**

The literature for this review was collected from PubMed, Scopus, Research Gate and Google Scholar.

**Result:**

The three APOE isoforms are APOE4, APOE3, and APOE2. APOE4 raises the risk of AD by causing earlier and more abundant amyloid pathology and impairs BBB integrity and several aspects of normal brain functions. APOE4 affects the production, clearance, and aggregation of Aβ, leading to amyloid plaque formation. It also has other effects, like increasing tau hyperphosphorylation to neurofibrillary tangle, neuroinflammation, and mitochondrial and synaptic dysfunctions.

**Conclusion:**

APOE4 contributes to AD pathogenesis, mainly through BBB integrity and pathways dependent on amyloid beta. Therefore, APOE4‐targeted therapy could be useful for Alzheimer's disease treatment.

